# A staffing perspective on barriers to and facilitators of temporary worker safety and health

**DOI:** 10.1002/ajim.23509

**Published:** 2023-07-10

**Authors:** Lauren M. Menger‐Ogle, Devin Baker, Rebecca J. Guerin, Thomas R. Cunningham

**Affiliations:** Division of Science Integration, National Institute for Occupational Safety and Health (NIOSH), Cincinnati, Ohio, USA

**Keywords:** barriers, facilitators, occupational safety and health, staffing industry, temporary workers

## Abstract

**Background:**

Research has documented occupational health disparities, including higher rates of work‐related injuries, among temporary workers compared with workers in standard employment arrangements. According to guidance from the Occupational Safety and Health Administration (OSHA) and the National Institute for Occupational Safety and Health (NIOSH), both staffing companies and host employers are responsible for protecting the occupational safety and health (OSH) of temporary workers. To date, there has been little qualitative research on temporary worker OSH in the United States and a lack of evidence‐based OSH programs designed to meet the needs of temporary workers. The aim of this study was to better understand the barriers to and facilitators of temporary worker OSH from the perspective of US staffing companies.

**Methods:**

In‐depth interviews were conducted with a convenience sample of representatives from 15 US staffing companies. Interviews were audio recorded, transcribed verbatim, and analyzed through a three‐step process.

**Results:**

Commonly mentioned barriers to temporary worker OSH include differential treatment of temporary workers by host employers; lack of understanding among host employers and staffing companies of joint OSH responsibilities; and workers’ fear of job loss or other negative repercussions if they report an injury or illness or voice OSH concerns. Commonly mentioned facilitators of temporary worker OSH include conducting client assessments and site visits and fostering strong communication and relationships with both host employers and temporary workers.

**Conclusions:**

These findings can help inform the tailoring of OSH programs to promote health equity in temporary workers.

## 1 | INTRODUCTION

Recent decades have seen a marked increase in labor market flexibility, in which the standard employment relationship (i.e., an employee works exclusively for one employer on a predictable schedule with the mutual expectation of long‐term employment) is being replaced by various nonstandard work arrangements, including temporary agency work.^1–3^ Temporary agency workers (from here on out referred to as “temporary workers”) are those who are hired by a staffing company to work at the site of a host employer company. In 2017 (the latest available data), there were an estimated 1.4 million temporary workers in the United States, which is equal to 0.9% of total employment.^4^ Previous studies have shown temporary workers tend to be paid less, receive fewer benefits, and are more likely to be younger and be persons of Hispanic ethnicity compared with workers in standard employment arrangements.^5^

Among temporary workers compared to workers in standard employment arrangements, there is increasing evidence of higher rates of occupational injuries,^6–11^ and greater burden of work‐related illnesses and psychological morbidity.^11,12^ Due to confusion over which employer is supposed to record temporary worker injuries and illnesses on their OSHA 300 Log^13^ and other limitations of the Survey of Occupational Injuries and Illnesses and Injuries, this national survey does not accurately capture temporary worker injuries and illnesses in the United States. As such, workers’ compensation data are currently the best way to assess temporary worker injuries and illnesses. Studies examining workers’ compensation claims in Washington State found temporary workers have a two‐fold higher claim rate compared to their non‐temporary peers.^8,10^ A recent study looking at workers’ compensation claims in Ohio also found elevated injury rates for temporary workers compared to permanent workers.^6^ Because temporary workers may underreport injuries and illnesses for fear of job loss or other retribution,^14–16^ it is likely the actual burden of occupational injuries and illnesses they experience may be higher than these studies suggest.

There is also evidence suggesting injuries incurred by temporary workers may be more severe and costly compared to those experienced by non‐temporary workers.^6,9,10,17^ In the previously mentioned study focused on workers’ compensation data in Ohio, temporary workers were found to have higher rates of severe injuries with 8 or more days away from work compared to nontemporary workers in most industry sectors.^6^ Similarly, in Washington state, temporary workers had 1.5 times greater median time loss per claim (40 vs. 27 days) compared to those in standard employment arrangements, suggesting a greater loss in productivity associated with temporary worker injuries.^10^ Another study in Minnesota found claim costs for temporary workers were three times greater compared with costs for regular full‐time employees, primarily due to a higher frequency of claims.^9^

Previous, but still limited, research has focused on identifying the underlying factors that contribute to temporary workers’ increased risk for work‐related injury and illness.^16^ Compared with workers in standard employment arrangements, temporary workers may be: assigned to more hazardous jobs; less familiar with work operations, associated hazards, and protective practices; and less likely to speak up about safety and health concerns.^14,18,19^ Temporary workers are also likely to be new hires multiple times a year, and newly hired employees have a higher risk of being injured at work compared to employees with longer job tenure.^20,21^ Due to their short job tenure, they may also lack a social connection to other workers who could help mitigate exposure to hazards in the workplace.^2^ Temporary workers are in a constant state of job insecurity^22^ and have frequent periods of unemployment, both of which have been established as chronic work‐related stressors^23–25^ that can negatively impact mental and physical well‐being.^26,27^ Employment and income insecurity may negatively influence safe work practices, making temporary workers more likely to accept hazardous tasks, cut corners, and work while injured.^28^ Lack of health benefits and paid sick leave may also contribute to the increased risk of occupational injuries and illnesses among temporary workers.^2^ Workers with paid sick leave have been shown to be 28% less likely to sustain a work‐related injury compared to workers without paid sick leave.^29^ Temporary workers may also be less likely to have pre‐assignment screening, be given appropriate safety equipment, or receive adequate safety and health training.^8,30,31^ Risk may also be heightened for temporary workers because of confusion as to which employer in the dual employment arrangement—the staffing company or the host employer—bears responsibliliy for various aspects of protecting the safety and health of temporary workers.^8^

In response to the high rates of occupational injuries among temporary workers, the Occuptional Safety and Health Administration (OSHA) launched its Temporary Worker Initiative (TWI) in 2013. Although staffing companies are legally responsible for paying wages, workers’ compensation, and unemployment premiums, host employers are considered joint employers of tempoary workers because they supervise and control the work. Therefore, according to OSHA, staffing companies and host employers are both responsible for protecting temporary workers’ safety and health.^32^ The OSHA TWI has issued a number of guidance documents outlining recommended practices for staffing companies and host employers “so they may better protect temporary workers through mutual cooperation and collaboration.”^32(p. 1)^ This guidance focuses on the joint OSH responsibilities of staffing companies and host employers with regard to risk assessment, contracting, communicaiton, training, personal protective equipment (PPE), and injury and illness response, recordkeeping, and prevention. The OSHA TWI has issued several bulletins on specific OSH topics, such as hazard communication and respiratory protection. Although the guidance issued through the OSHA TWI is an important step toward protecting and promoting the OSH of temporary workers, evidence‐based interventions are needed to further protect this vulnerable population of workers. Effective interventions should be tailored to the unique challenges, needs, and realities of temporary workers and the staffing industry to ensure program adoption, effectiveness, and sustainability.

Research on temporary worker OSH, is still in its infancy, and a majority of the work that has been conducted thus far has been quantitative, rather than qualitative, in nature.^6–11,22^ Existing qualitative research has focused on understanding temporary worker OSH from the perspective of temporary workers and host employers.^8,16,33,34^ However, to our knowledge, there has been only one published qualitative study focused on the barriers to and facilitators of temporary worker OSH from the perspective of staffing companies. Underhill and Quinlan^35^ conducted focus groups with staffing company and host employer representatives in Australia to identify promising approaches for protecting temporary workers and to explore ways to improve regulatory compliance and inform future policy interventions. Similar studies are needed to determine similarities and differences in OSH barriers and facilitators as perceived by staffing companies. To fill this gap, in‐depth interviews were conducted with representatives from U.S. staffing companies. The research questions addressed through this study were as follows:

1. Do US staffing company representatives perceive temporary workers to be at a higher risk of being injured at work compared to non‐temporary workers? If so, why?
2. What do US staffing company representatives perceive as the barriers to and facilitators of protecting temporary worker OSH?

## 2 | MATERIALS AND METHODS

### 2.1 | Recruitment

A convenience sample of representatives from US staffing companies was recruited to be interviewed through two partner organizations: a professional association and a university‐based public health program. The professional association recruited participants who were involved in a safety committee led by the association, and the university‐based public health program recruited participants who had attended or expressed interest in attending an OSH training the program was offering for staffing companies. These two partner organizations recruited representatives for whom temporary worker OSH was their main job responsibility or one of their primary responsibilities. Staffing companies that solely place temporary workers in finance, public administration, and professional and business services jobs were excluded due to the minimal OSH risks in these occupations. Recruitment continued until saturation of themes was obtained, and additional new themes no longer surfaced during interviews.^36^ Of the 18 representatives from 18 different staffing companies who were contacted by email, 15 agreed to participate in an interview (83.3% response rate). Two of the companies elected to have more than one employee participate in the interview (one company had two representatives and another company had three representatives).

### 2.2 | Interview procedure

All interviews were conducted by the first author by phone and lasted between 43 and 105 min (mean = 78.3 min, standard deviation [SD] = 17.16 min). Before starting the interview, participants were asked to give verbal consent to participate because no identifying information was otherwise being collected. After providing consent, participants were asked a series of background questions related to their role and tenure as well as the size of their staffing company and the types of industries within which their company places temporary workers. For the two interviews with more than one employee, background information was only collected from the person who identified themselves as the primary interviewee. Interviews were conducted using a semi‐structured interview guide with flexibility to allow for the emergence of other topics relevant to temporary worker OSH. Participants were asked to describe their perceptions related to temporary worker OSH risks as well as barriers to and facilitators of protecting and promoting the safety and health of temporary workers. Example questions include:

- “*Do you feel temporary employees have different health and safety issues or concerns compared to non*‐*temporary employees? If so, please describe.*” (Risk perceptions)
- “*What barriers or challenges are there to promoting workplace health and safety among temporary workers?*” (Barriers)
- “*What are some key strategies or best practices when it comes to helping temporary workers be safer and healthier on the job?*” (Facilitators)

Participants were also asked to describe their staffing company’s current OSH practices and perceptions on OSH training needs within the staffing industry; these results are currently being analyzed and will be reported elsewhere. All interviews were audio recorded and transcribed verbatim. The [Institution name removed to facilitate blind review] Institutional Review Board reviewed and approved all study materials and procedures.

### 2.3 | Participants

Most participants were from safety/risk management (66.7%), three were from Human Resources (20%), and two were executive level (13.3%). Time working in the staffing industry ranged from 1.5 years to 28 years (median = 14 years). The participating staffing companies were based across all regions of the United States, and ranged in size in number of internal employees (range = 12–6000, median = 140), the number of temporary employees on the payroll at any given time (range = 220–350,000, median = 2,500), and the number of host employer clients to whom they provide temporary workers at any given time (range = 20–200,000, median = 850). Each company had between one and 26 employees dedicated to OSH as part of their core job description (median = 3). Participants indicated their staffing companies place temporary workers in a variety of industries, most commonly manufacturing (73.3%); transportation, warehousing, and utilities (66.7%); and services (60%). See Table 1 for additional descriptive characteristics of participants and the staffing companies they represent.

### 2.4 | Data analysis

Data analysis was conducted in three stages guided, in part, by the framework outlined by Braun and Clarke.^37^ First, two members of the research team independently conducted open coding of each transcript and met to discuss and generate an initial list of jointly identified themes. Discrepant opinions were discussed until consensus was achieved, and each theme operationally defined. One member of the research team then applied the codes to all transcripts, and a second member of the research team applied the codes to one‐third of the transcripts (*n* = 5) that were selected using a random number generator. A preliminary analysis of these five coded transcripts revealed minimal, additional discrepancies, which the two coders discussed until consensus was achieved. Finally, inter‐coder agreement was calculated based on the five transcripts that were coded by two members of the research team, resulting in a Holsti Index of 80.6% agreement. The Holsti Index, a widely used metric of inter‐coder agreement, reflects the percentage of agreement in coding decisions out of the total number of coding decisions made by the two coders.^38^ Eighty percent is considered an acceptable level of agreement.^38^ All analyses were conducted using ATLAS.ti software.^39^ The themes are organized by the research questions.

## 3 | RESULTS

### 3.1 | Risk perceptions

When asked if they believed temporary workers are at a higher risk of being injured at work compared to non‐temporary workers, ten participants said yes (66.7%), one participant said no (6.7%), and two participants said it depends (13.4%; two participants did not voice an opinion). Of those who said yes, common perceptions as to why temporary workers may be at a higher risk of being injured at work include dual employment (e.g., the inability of staffing companies to oversee the day‐to‐day work of the employee); assignment to more dangerous jobs and tasks compared to non‐temporary workers; frequency with which temporary workers are new to the job; and not receiving sufficient OSH training.

*I think that a lot of times the temporary associate is used for work that either is adverse risk, you know, risk transfer almost where a client might not want to have his own permanent people doing a specific task so they’ll call in the temp agency to do it*. (#5)

*I see a lot that temporary workers aren’t given the same in‐depth safety training that their current employees would be, and it puts them at risk for injury a lot more often*. (#15)

Two of the participants who said yes to the questions of whether they perceived temporary workers are at a higher risk of being injured at work expressed an opinion that the data related to temporary worker injures may underrepresent the actual number of injuries incurred by temporary workers:

*There’s lots of other injuries that happen that there’s no data or statistics on. And a very small number of injuries are recordable. I mean, I keep a log with everything on it and then we sort through the ones that rise to the level of being recordable and so, anyway, there’s just a lot more happening out there than I think is understood*. (#14).

One of the participants, who said it depends, elaborated that temporary workers may be at higher risk of being injured if they are employed by a staffing company or host employer that does not do enough to protect temporary worker OSH.

*It depends on the client and it depends on the staffing firm that they’re working for. I think they can definitely get put into bad situations if it’s either a [host employer] company that doesn’t care about their temporary employees that they’re bringing, you know, that they’re having the staffing service provide or on the other side that the staffing firm doesn’t really care about safety and will place their employees just about anywhere*. (#4)

### 3.2 | Barriers to protecting temporary worker OSH

Participants described numerous barriers or challenges to protecting and promoting temporary worker OSH, including barriers pertaining to host employers, the nature of the staffing industry, and characteristics of the workers.

#### 3.2.1 | Host employers

Barriers pertaining to host employers included differential treatment of temporary workers; lack of OSH knowledge and awareness; deception; and making changes to temporary worker jobs or tasks.

##### Differential treatment of temporary workers

All 15 participants mentioned differential treatment of temporary workers on behalf of host employers as a barrier to temporary worker OSH. Examples of differential treatment include assigning temporary workers to more dangerous jobs; giving temporary workers less OSH training and fewer opportunities to participate in company safety programming compared to non‐temporary workers; and discouraging temporary workers from reporting injuries.

*The host employer considers the folks that get sent to them on a temporary basis as less than equal to their own employees. So, it’s kind of like a disposable commodity*. (#3)

Some participants thought this differential treatment might result from a belief among host employers that temporary workers are not worth the investment of their time and resources because of their short job tenure.

*You’re trying to get the client to commit to training and all that, and in their mind it doesn’t make sense for them to put that type of time and effort in because they know the employee’s only going to be there 20‐something days*. (#5)

##### Lack of OSH knowledge and awareness

A majority of participants (*n* = 12) mentioned host employer lack of knowledge and awareness about OSH—both in general and specifically in the joint responsibilities of staffing companies and host employers related to temporary worker OSH as specified by the OSHA TWI—as a barrier.

*There are still a lot of customers that I get the deer in the headlight look when I go “Well, you know about the OSHA Temporary Worker Initiative started back in 2013,” and they just stand there and look at you and they don’t. They don’t know*. (#2)

##### Deception

Participants (*n* = 9) also mentioned deception by host employers, such as making false promises to take certain measures toward protecting temporary worker OSH and not being fully transparent about the hazards temporary workers would be exposed to at the worksite, as another barrier to temporary worker OSH.

*One of our postaccident investigation questions, and we’ll talk directly with the employees, is, you know, tell us about the training that you received before you started. And it’s amazing sometimes between what we were told during the worksite evaluation period and what the temp actually experienced, what a huge difference there is*. (#5)

##### Job change

According to two‐thirds of the participants (*n* = 10), the issue of job change is another barrier to temporary worker OSH. This occurs when a host employer makes changes to a temporary worker’s job or tasks without first informing the staffing company and ensuring the worker has the relevant qualifications and experience, or that the worker has the training or PPE needed to complete the new job or task safely.

*You know, maybe they were put out there to be a machine operator but now the client has them operating a forklift…and that would be a great situation where we would go in and say “Okay, we said we would provide you this, now you have them doing this…and you haven’t told us and you haven’t properly screened them for that particular job or trained them for that job*. (#4)

Some participants (*n* = 6) perceived the root cause of this issue as a breakdown in communication between the staff at the host employer who are either supervising and controlling the work of temporary workers and the staff at the host employer who are negotiating the contracts (often someone in Human Resources).

*We have communicated that with the [host employer] contact in the main office when we sign the contract and we’ve worked out all the details of working together. But the chance of that information getting down to his 40th manager down the line is slim to none, and so there’s a huge communication gap between, you know, who we initially talk to about safety and that frontline manager over the temporary [worker]*. (#9)

#### 3.2.2 | Staffing companies

Barriers to temporary worker OSH pertaining to staffing companies included limited power and control; prioritizing business goals over OSH; a lack of OSH knowledge of behalf of staffing company employees; and a lack of time and resources for OSH. Participants speculated that there may be differences in these barriers by company size.

##### Limited power/control

One barrier mentioned by participants (*n* = 9) is the limited level of power and control staffing companies have over OSH. Because they often do not have full‐time staff onsite at their host employer clients’ worksites, they are not able to observe the workers and they have little to no control over the work environment or how the work is done.

*Because we’re not at the client’s site, we don’t live [in] their world and we don’t know the specifics of exposures, you know. That makes it a challenge*. (#11)

*I think one of the biggest challenges that we face in the staffing industry is that we can do everything in the world…to promote safety and to educate our associates on safety. But at the end of the day, we can never overcome our customer’s safety program. It’s their program, their culture is always going to prevail*. (#1)

##### Prioritizing business goals over OSH

Participants (*n* = 9) mentioned the fear of losing business for placing too many OSH demands on their host employer clients as another barrier to temporary worker OSH.

*But the reality is we get pushback from our clients to say, you know what, if you’re going to be too restrictive with us, we’ll just go to your competitors down the road. Because they’re not going to lecture us. They’re not going to make us do this, this, and this*. (#6)

##### Lack of OSH knowledge and awareness

In addition to host employers’ lack of OSH knowledge and awareness, participants (*n* = 8) also mentioned a lack of OSH knowledge and awareness on behalf of staffing companies as a barrier to temporary worker OSH. This included a lack of knowledge related to OSH in general, the joint OSH responsibilities of staffing companies and host employers, and the hazards and protective factors associated with the jobs in which they are placing temporary employees.

*I think the problem that’s more in the line of the staffing industry is there are so many staffing companies out there that just‐ they don’t have the general awareness piece down. They don’t even look at sites, and it’s just like “We need a forklift operator tomorrow.” “Okay, we’ll have somebody there for you.”* (#2)

##### Lack of time and resources for OSH

Participants (*n* = 8) mentioned a lack of time and resources, including not having enough staff to conduct host employer site visits and not having OSH training materials tailored for the staffing industry, as another barrier to temporary worker OSH.

*Whether it’s follow‐up directly with the employees instead of waiting for someone to call us with a concern…[or] it’s randomly stopping into a client and watching them work. There’s a lot more that I think, you know, our staffing agency, and probably many others can do once the employee walks out the door. Like our responsibility does not end the second, you know, they leave. And I realize that, you know, we don’t always have the manpower to do that*. (#5)

Participants also noted time to provide general OSH training to temporary workers is limited due to pressure from host employers to fill orders quickly. Furthermore, temporary workers’ often have a limited attention span for OSH training, particularly given all the other aspects of new worker orientation (e.g., completing background checks, tax forms, and other paperwork) that require their time and energy.

##### Variation by company size

Participants (*n* = 6) speculated that there may be differences in barriers based on staffing company size. For instance, small staffing companies may have less OSH knowledge, resources, and programming compared to larger staffing companies (e.g., smaller staffing companies may not have a dedicated safety professional and may not have access to OSH resources through their workers’ compensation carrier or professional associations).

*The small mom and pop shops that I feel that OSHA is really targeting as the staffing industry, they don’t have the resources that‐ that we have*. (#1)

*I’ll admit to you there are some players out there that aren’t so big. In fact, I was at one yesterday giving them a class because they wanted a class in risk management, so we accommodated. And they don’t really have a structured safety. They have a guy. They say, “Hey, you’re the safety guy,” but he’s got no background [in OSH]*. (#8)

One participant argued that alternatively, because they represented a smaller company with fewer clients and temporary employees on the payroll, they are able to devote more time and attention to OSH for each individual client and temporary worker assignment than would be possible for a larger staffing company. For instance, they are able to conduct a site visit to assess the safety conditions for each new client, which is something a larger staffing company may not have the manpower to do.

#### 3.2.3 | Worker characteristics

Participants (*n* = 11) mentioned several ways in which various characteristics of temporary workers may pose as barriers to protecting their safety and health at work. For instance, participants indicated that temporary workers may not view OSH as important because of their short job tenure, or they may fear job loss or other negative repercussions if they report an injury or illness or voice concerns about safety. Nine participants indicated host employers sometimes end up directly hiring temporary workers when their temporary assignment ends. Some of these participants (*n* = 4) stated fear of ruining chances of being hired on full‐time by a host employer as another barrier that might prevent temporary workers from reporting an injury or speaking up about an OSH issue. Participants also mentioned that temporary workers tend to be young and inexperienced and, as a result, have limited knowledge and awareness about OSH hazards and protective measures. Another theme that arose was that temporary workers may prioritize earning money to support themselves and their families and, as a result, may be willing to take on more risky jobs or exaggerate their skills and abilities to get a job they are not qualified for.

*They’re not going to say anything because the most important thing to that individual is the money…they will do whatever it takes and they will say nothing because they want to get hired on by that customer in many cases… and they feel if they say something, the customer will, you know, tell them to leave and they won’t be able to go back*. (#3)

People from different cultures who are not familiar with U.S. safety standards or have a culture of not speaking up to authority figures was also mentioned as a worker characteristic that may pose a barrier to temporary worker OSH.

### 3.3 | Facilitators of protecting temporary worker OSH

Participants were asked to describe what they viewed as key strategies or facilitators of protecting and promoting the OSH of temporary workers. Some of the most frequently mentioned facilitators include client assessments and site visits; host employers that value OSH and treat temporary workers the same as non‐temporary workers; communication and relationship building; training; injury and illness reporting and response procedures; the OSHA TWI; and provision of OSH assistance to host employers.

#### 3.3.1 | Client assessments and site visits

Participants (*n* = 9) mentioned client assessments and site visits as facilitators of temporary worker OSH. Whether as part of an initial client assessment or on an ongoing basis for existing clients, site visits allow the staffing company an opportunity to jointly review the job description, hazards, and protections in place with the host employer; to observe the workers and the work environment; and to see the host employer’s safety program in action.

*I think‐ you know, I think the key to keeping the temporary employee safe is‐ I think it always starts with the clients that you’re bringing in and you have to have a good process of evaluating those clients to make sure that they’re going to provide a safe workplace for our employees*. (#4)

Client assessments may also include reviewing OSHA 300 Logs, safety program materials, and other documents. Some participants mentioned using OSH checklists during client assessments and site visits and the importance of talking with those who will be supervising the work of temporary workers while on site.

#### 3.3.2 | Host employer treatment

Host employers that value OSH, have a strong safety culture and a comprehensive safety program, and treat temporary workers the same as non‐temporary was also mentioned by participants (*n* = 9) as a facilitator of temporary worker OSH.

*I think it comes down to just the character of the [host] employer. You know, if they truly care. What’s their reasoning behind why they’re doing it? Do they truly care? Or do they just want to ‐‐ hey, we’re just ‐‐ we just don’t want to get a fine*. (#7)

Some participants provided examples of how they try to encourage these practices among their host employer clients, for example:

*My mantra is that every one of these temporary associates are the most important person in someone’s world and, you know, you have to treat them as such. So, if you wouldn’t do this to the most important person in your world, why are you doing it to someone else?* (#3)

#### 3.3.3 | Communication and relationship building

Frequent communication and relationship building with temporary workers and host employers were also mentioned by participants (*n* = 9) as facilitators of temporary worker OSH. Participants utilize many different methods of communication, from in‐person meetings, phone calls, emails, and text messages, to newsletters and posters displayed at both the staffing company and host employer sites. Participants noted the importance of following up with temporary workers shortly after they have started a new assignment to make sure they have been provided with necessary training and PPE, to remind them to report safety problems or concerns to both the staffing company and the host employer, and to ensure they have not been asked to complete any tasks outside of those they were contracted to perform. Beyond just communicating with temporary workers, participants also noted the importance of building relationships to make them feel more comfortable speaking up about OSH problems or concerns.

*You’ve got to talk to them [temporary workers], you’ve got to build a relationship with them first, let them know you care, that they’re not going to get in trouble for anything they say, but that they can trust…You need to build a relationship with them as a person, okay? And then you can ask them questions about their workday, and they’re more willing to share that.* (#7)

One participant mentioned use of a pocket card, with a statement about temporary workers’ stop work authority (i.e., their responsibility and authority to stop work if they feel it is unsafe) and a place for the worker to sign indicating their commitment to safety, as an effective means of keeping safety top of mind and empowering workers.

In discussing communication with host employers, participants noted the importance of holding regular meetings to discuss OSH issues and communicating directly with those who are supervising and controlling the work of temporary workers.

*My favorite safety partnerships are the ones where I meet with them regularly and we talk about all these things and that way I have an ongoing opportunity to educate them*. (#14)

#### 3.3.4 | OSH training

OSH training—including general awareness OSH training as well as site‐ and task‐specific OSH training for temporary workers—was also mentioned by participants (*n* = 8) as a facilitator of temporary worker OSH. Participants emphasized the importance of having workers complete a post‐training knowledge assessment to ensure their understanding of the training content and obtaining documentation of training provided by both both the staffing company and the host employer to increase accountability.

*I believe that documented training is much more effective training. If I come up to you and I say “Hey, you’re going to train our people on that equipment, right?” “Oh yeah, sure I am.” “Do you mind sending me the documentation or signing this piece of paper?” “Oh, well, yeah I’ll do that.” What’s going to happen to that employee? They’re probably going to be much better trained because [that person] put her name on a piece of paper saying she trained them*. (#2)

Participants also noted the importance of providing OSH training to internal staffing company employees to ensure they are aware of and can better protect temporary workers from the hazards they face on the job.

*I think education is truly the key to injury prevention and I think it’s education in every aspect from whether you’re a recruiter or whether you’re a temporary employee*. (#12)

#### 3.3.5 | Injury and illness reporting and responding procedures

Participants (*n* = 8) mentioned various aspects of injury reporting and response procedures as keys to facilitating temporary worker OSH. They emphasized the importance of encouraging host employers to report to the staffing company if they see a worker do something unsafe, encouraging workers to report to both the staffing company and the host employer when they have a safety concern or experience a work‐related injury or illness, and following‐up with the host employer after a worker has reported a concern to ensure the needed steps are being taken to protect workers from the hazard. Strategies used to reduce workers’ fear of reporting include encouraging workers to report all safety concerns and injuries even if they perceive them as minor and assuring workers that they will not lose their job for reporting. Other strategies mentioned were providing workers with a list of contacts, so they know who to call and providing positive reinforcement when workers do report an injury or concern.

*We do start talking to people about accident reporting and you know, the idea that even if you think it’s minor, you think it’s okay don’t need medical treatment that you know, even if you don’t want but that’s expected as part of your job to report that to the supervisor at the site as well as contacting us*. (#10)

Participants also emphasized the importance of jointly conducting incident investigations with host employers, talking with temporary workers as part of such investigations to solicit their ideas for how to prevent similar incidents from happening again in the future, and retraining workers if necessary.

*So, part of our accident investigation, we actually ask people…”How could you have prevented this from happening?” And sometimes they come up with some really simple ideas that don’t cost anything or have‐ you know, are really low‐cost that we share with our clients*. (#13)

One participant said if they receive a report of a safety issue or concern, they will do “quality control” calls to other temporary workers at the same site to see if others are also having OSH problems.

#### 3.3.6 | OSHA TWI

The OSHA TWI was also mentioned by participants (*n* = 8) as a facilitator of temporary worker OSH. Specifically, participants view the guidance issued through the TWI as valuable in clarifying the joint OSH responsibilities of staffing companies and host employers and useful as a tool for educating host employers.

*And I’ll say that the launch of the Temporary Worker Initiative was music to my ears and has been so helpful in kind of explaining to the host employers what their specific obligations are with respect to health and safety training*. (#12)

#### 3.3.7 | OSH assistance

Participants (*n* = 7) mentioned providing various forms of OSH assistance to host employers as another facilitator of temporary worker OSH. Assistance may include helping with the development of training materials, evaluating and helping to improve host employer safety programs, and sending safety consultants and specialists to host employer sites to help further improve their safety programs.

*I take her [safety consultant] out there to my biggest clients with me and she does a walkthrough of their building‐ in some cases I take her in up front before we’ve even signed a contract, in other cases I bring her in later just as a courtesy to the client. And she walks the plant and she does a written analysis of what she finds, which helps because that’s a little easier coming from her than it is from me*. (#14)

## 4 | DISCUSSION

The purpose of this study was to conduct a qualitative investigation of perceptions related to temporary worker OSH risks and barriers to and facilitators of protecting temporary worker OSH from the perspective of a convenience sample of US staffing company representatives. Most participants believed temporary workers are at a greater risk of being injured at work compared to non‐temporary workers. Participants noted numerous barriers to temporary worker OSH pertaining to host employers, including differential OSH treatment of temporary workers compared with non‐temporary workers and not being fully open and honest when communicating with staffing companies about OSH hazards and OSH programming for temporary workers. Hopkins^34^ conducted in‐depth qualitative interviews with first‐line managers and human resources managers from host employers, as well as with permanent and temporary workers (including temporary workers directly employed by the host employer and those employed through a staffing company), and found temporary workers hired through a staffing company received insufficient safety and health training and PPE compared with their permanent and directly‐hired temporary worker peers. According to OSHA, in most cases, staffing companies are responsible for providing temporary workers with general awareness OSH training, and host employers are responsible for providing site‐ and task‐specific safety and health training.^32^ Site‐ and task‐specific training should be identical or equivalent to the training provided to non‐temporary workers doing the same or similar tasks.

Another identified barrier is when a host employer makes changes to a temporary worker’s jobs or tasks, outside of those they were contracted to perform, without first informing the staffing company. Undisclosed job changes can put temporary workers at increased risk of being injured because they may not have the necessary skills or qualifications to do the new job, and they may go without the additional training and PPE needed to do the new job safely Foley^8^ conducted workers’ compensation case follow‐up interviews with injured temporary workers in Washington State and identified the issue of job change as a common theme that contributed to temporary worker injuries. Some participants in the current study believed undisclosed job changes may be due to a breakdown in communication, in which the frontline supervisors at the host employer are not well‐informed about what the assigned tasks are and the requirements to inform the staffing company of changes to those tasks, regardless of whether these stipulations are stated in the written contract.

Lack of OSH knowledge in general, and specifically the joint responsibilities as stated in the OSHA TWI guidance was mentioned as a barrier that pertained to both host employers and staffing companies. Other identified barriers pertaining to staffing companies include their limited power and control over the work environment and the work itself; prioritizing business goals over OSH; and an overall lack of time and appropriate resources for OSH. Participants noted there are likely differences in these barriers between smaller and larger staffing companies; for instance, smaller staffing companies may have particularly limited resources for OSH programming. Other studies have found that workers in small companies report receiving less safety training compared to workers in larger companies.^40^

Characteristics of temporary workers, such as fear of speaking up and prioritizing earning money over OSH, were also mentioned as barriers. These findings have been corroborated by other qualitative studies.^8,16,33,35^ Foley^8^ also conducted unpublished field interviews with staffing agency managers and host employers and found that temporary workers’ desire to become permanent employees may act as an incentive to under‐report injuries and avoid speaking up about safety and health concerns. In a different but complementary study, Luria and Yagil^41^ found that temporary workers have more of an individual‐focused view of safety, whereas non‐temporary employees have more of a group‐ or organizational‐focused view of safety. In other words, temporary workers are less likely to believe their organization can or will protect them, so they feel as though they have to protect themselves. Participants in the current study also suggested temporary workers tend to be young and inexperienced, which has also been noted by other studies,^5,6^ and as a result they may have limited knowledge and awareness about OSH hazards and protective measures. Taking all of these findings into consideration unveils a complex web of factors that may influence temporary workers’ safety knowledge, attitudes, behaviors, and willingness to speak up.

Identifying facilitators of temporary worker safety and health from the perspective of staffing industry representatives in the United States is a particularly novel contribution of this study. The facilitators most often mentioned by participants include conducting client assessments and site visits; providing OSH training for temporary workers and internal staffing company employees; and fostering frequent communication and relationship building between staffing companies, host employers, and temporary workers. Underhill and Quinlan^35^ also identified the importance of building long‐term relationships between staffing companies and host employers, staffing companies conducting regular worksite visits, and staffing companies following up with temporary workers after the start of an assignment to ensure they have received the necessary training and that their job assignment hasn’t been changed, as facilitators of temporary worker OSH.^35^ In the previously mentioned case follow‐up interviews with injured temporary workers in Washington State, Foley^8^ also highlighted the importance of improving the frequency and adequacy of OSH training for temporary workers as well as having staffing companies conduct ongoing monitoring of host employer safety practices as important facilitators of temporary worker safety and health. Participants also mentioned improving awareness and adoption of the OSHA TWI guidance by staffing companies and host employers; beneficial aspects of staffing company injury and illness reporting and response procedures (such as following up with the host employer after a worker has reported a safety or health concern); and staffing companies providing various forms of OSH assistance to host employers (such as assisting them with the development of OSH training materials) as facilitators of temporary worker OSH.

Though many of the findings from the present study align with those of Underhill and Quinlan’s^35^ qualitative focus group study with host employers and staffing companies in Australia, there were some notable differences. For instance, in the present study, some of the most commonly mentioned barriers related to host employers included host employers’ differential OSH treatment of temporary workers compared to non‐temporary workers and perceived lack of transparency when divulging the hazards and protections for temporary workers to staffing companies. These barriers were perceived to be true regardless of company size. Underhill and Quinlan^35^ found more of an emphasis on the role of company size, suggesting it was primarily smaller host employers and staffing companies that had limited OSH knowledge and resources and a limited commitment to OSH. Underhill and Quinlan^35^ also identified a recurrent theme on the value of a niche provider approach for variations in facilitators, in which staffing companies specialize in providing a specific industry that they are knowledgeable about; a theme that did not surface in the present study.

### 4.1 | Practical implications

The findings from this study provide unique insight into ways staffing companies can facilitate temporary worker OSH. Staffing companies can enhance their efforts to conduct client assessments and ongoing site evaluations; provide high‐quality general awareness OSH training to temporary workers as well as to their internal staffing company employees; and maintain frequent communication and build strong relationships with both temporary workers and host employers. Staffing companies can also encourage host employers to give temporary workers the same OSH training and PPE that is provided to non‐temporary workers doing the same work and to communicate with staffing companies before making a change to a temporary worker’s job assignment, so both employers can ensure any additional training and PPE is provided. Staffing companies can follow‐up with temporary workers shortly after they begin a new assignment to ensure they have received adequate site‐ and task‐specific training from the host employer and to ensure they have not been asked to perform any tasks outside of their job description. If a worker is injured, staffing companies should work together with host employers and the affected worker(s) to investigate the incident and develop a plan to prevent similar incidents from occurring in the future. All the mutually agreed upon OSH responsibilities of the staffing company and host employer can be documented in the written contract between the two firms, and reviewed regularly, to ensure clarity and accountability. Staffing companies and host employers can also work together to ensure those who are supervising the work of temporary workers are briefed on these responsibilities.

It also is important for both staffing companies and host employers to understand and target the many factors that influence the safety and health views and behaviors of temporary workers. Both employers should strive to create an environment in which temporary workers know how to report work‐related injuries and illnesses, near‐misses, and OSH concerns and feel encouraged and comfortable doing so. This may be particularly important for workers from racial and ethnic minority groups.

### 4.2 | Policy implications

There is a clear need to raise awareness among both staffing companies and host employers about the OSHA TWI guidance on the joint safety and health responsibilities of both parties. There is also a need for resources outlining OSH best practices for staffing companies and host employers in more detail than is currently available in the OSHA TWI resources. Toward this aim, NIOSH‐in collaboration with the National Occupational Research Agenda (NORA) Services Sector Council, the American Staffing Association, and other partners‐recently published an in‐depth set of OSH best practices for host employers (https://www.cdc.gov/nora/councils/serv/protectingtemporaryworkers/host-employers.html?s_cid=3ni7d2-Manuscripts_PTW_2022).^42^ This document includes checklists (https://www.cdc.gov/niosh/docs/2022-126/2022-126_Checklists_508.pdf) that can be used to help host employers ensure they are going beyond compliance to protect the safety and health of temporary workers. There is also a complementary slide deck (https://www.cdc.gov/nora/councils/serv/protectingtemporaryworkers/docs/NORA-HE-Doc-Slide-Deck-for-Staffing-Companies_final.pptx) staffing companies can use to educate their host employer clients about the best practices. A similar set of OSH best practices for staffing companies will be forthcoming.

There are currently no federal regulations specific to OSH for temporary agency work in the United States. A small number of states have passed legislation in recent years related to temporary worker OSH, such as a 2013 rule in Massachusetts requiring staffing companies to provide temporary workers with a complete job description, information about required PPE, and other basic information about the job they are being hired to perform before beginning an assignment.^43^ More recently, Washington State passed a bill mandating communication between staffing companies and host employers over training and hazards and requiring staffing companies to withdraw their workers from sites where hazards have not been addressed.^44^

### 4.3 | Limitations

There are limitations that should be considered when interpreting the findings from this study. One limitation is the small sample size. However, repeated themes were identified across the transcripts indicating that saturation was reached.^36^ It is possible that participants were influenced by social desirability during the interview, given the research was conducted by NIOSH, a government agency. Although efforts were made to recruit representatives from staffing companies of various sizes, the median number of internal employees (*n* = 140) and the median number of temporary workers employed at any given time (*n* = 2500) suggest the convenience sample obtained primarily consisted of representatives from larger staffing companies. As previously noted, smaller staffing companies may have limited resources for OSH, including not having dedicated and well‐trained OSH professionals and, therefore, may have different perspectives on the barriers to and facilitators of temporary worker OSH. Smaller staffing companies may also be more motivated by a sales perspective and, as such, may not be as likely as larger staffing companies to encourage host employers to make investments in the safety and health of temporary workers. In addition, the participants who agreed to be interviewed may represent staffing companies with more of a focus on safety than other staffing companies since they were either members of a safety committee run by a professional organization, or they had attended or expressed interest in attending an OSH training for staffing companies.

### 4.4 | Future research

Additional research is needed to understand the prevalence of the barriers identified in this study and how they may be interrelated to one another. Similarly, future research is needed to better understand the identified facilitators of temporary worker OSH and how they can be better promoted by both staffing companies and host employers. Additional research is also needed to better understand the differences in OSH barriers and facilitators between smaller and larger staffing companies so interventions can be tailored accordingly. Because temporary workers, staffing companies, and host employers all have different ideas and perspectives on barriers to and facilitators of temporary worker OSH, further research is needed with all three of these groups to fully understand the best means of protection. There is also a need to examine the relationship between the barriers and facilitators identified in this study and temporary worker OSH outcomes, such as injuries, illnesses, and near misses. Future research should also explore how other social determinants of health or intersecting identities (such as age, race, and ethnicity) may interact with temporary worker status to affect OSH outcomes.

Overall, there is a need to develop evidence‐based interventions to promote the safety and health of temporary workers. Interventions need to consider the underlying factors that contribute to increased risk for temporary workers. One model that has been used to explain these underlying factors is Quinlan and Bohle’s Pressures, Disorganization, and Regulatory (PDR) model.^28,45^ This model consists of three underlying factors that influence the OSH of temporary workers, and precarious workers more generally—economic and reward pressures; disorganization at the workplace; and regulatory failure. The PDR model has been used to explain poorer OSH outcomes among temporary workers in Europe.^46,47^ All three of these factors, some of which may be difficult to change given the nature of the industry, need to be considered when developing and implementing interventions to comprehensively address the determinants of temporary worker OSH.

This study also uncovered a need to develop evidence‐based OSH training resources that are tailored to the unique needs of temporary workers and the staffing industry. This training should be rooted in established health behavior theories and designed to promote OSH motivation and efficacy, rather than solely focusing on knowledge transfer, as knowledge is necessary but not sufficient on its own to bring about sustainable behavior change.^48^ Evidence‐based training resources are also needed for frontline supervisors of temporary workers to ensure they understand the scope of contracted tasks the temporary workers are permitted to perform and how to effectively communicate with temporary workers about OSH and make them feel comfortable voicing OSH incidents and concerns. Whether for temporary workers or their on‐site supervisors, training interventions should incorporate training transfer best practices^49^ and be evaluated using established methodological criteria.^50^

### 4.5 | Conclusions

The findings from this study provide valuable insights into the barriers to and facilitators of temporary worker OSH as perceived by a convenience sample of US staffing company representatives. These findings, taken into consideration with those from other previous quantitative and qualitative studies focused on temporary worker OSH, can be used to help inform the development of interventions to protect and promote the safety and health of temporary workers. Given temporary workers are a substantial segment of the workforce, both in the U.S. and abroad, efforts to improve their safety and health have significant global public health implications.

## Figures and Tables

**TABLE 1:** Descriptive characteristics for participants and the staffing companies they represent (*N* = 15).

Participant characteristics	Range	Median	Mean	*SD*
Years in position	1.5–18	4.00	6.65	6.04
Years with company	1.5–28	10	10.88	8.44
Years in staffing industry	1.5–28	14	13.05	7.96
Position	*N*	*%*		
Executive level	2	13.3		
Human resources	3	20		
Safety/risk management	10	66.7		
Region based In				
Midwest	1	6.7		
Northeast	3	20		
South	5	33.3		
West	6	40		
**Company characteristics (# missing)**	**Range**	**Median**	**Mean**	***SD***
Number of internal employees	12–6000	140	967.5	1675.4
Number of internal employees dedicated to OSH (3)	1–26	3	6.8	7.5
Number of temporary employees*	220–350,000	2500	37,121.3	90,023.1
Number of host employer clients* (2)	20–200,000	850	17,598.5	54,980.2
Primary industries served	*N*	*%*		
Agriculture, Forestry & Fishing	2	13.3		
Construction	4	26.7		
Healthcare and Social Assistance	1	6.7		
Manufacturing	11	73.3		
Oil and Gas Extraction	1	6.7		
Services	9	60		
Transportation, Warehousing & Utilities	10	66.7		
Wholesale/Retail Trade	2	13.3		

*Note*: Number of missing responses are in parentheses. Number of temporary employees and number of host employer clients are estimates for the number at any given time rather than the total annual number. Participants could select more than one primary industry served.

Abbreviations: *N*, number; OSH, occupational safety and health; SD, standard deviation.

## Data Availability

The data that support the findings of this study are available on request from the corresponding author. The data are not publicly available due to privacy or ethical restrictions.
